# Preventative Effects of Milk Fat Globule Membrane Ingredients on DSS-Induced Mucosal Injury in Intestinal Epithelial Cells

**DOI:** 10.3390/nu16070954

**Published:** 2024-03-26

**Authors:** Erica Kosmerl, Celeste Miller, Rafael Jiménez-Flores

**Affiliations:** Department of Food Science and Technology, The Ohio State University, Columbus, OH 43210, USA; kosmerl.3@buckeyemail.osu.edu (E.K.); miller.10212@buckeyemail.osu.edu (C.M.)

**Keywords:** mucin, milk fat globule membrane, phospholipids, in vitro digestion, cell culture, HT29-MTX, scanning electron microscopy

## Abstract

The goblet cells of the gastrointestinal tract (GIT) produce glycoproteins called mucins that form a protective barrier from digestive contents and external stimuli. Recent evidence suggests that the milk fat globule membrane (MFGM) and its milk phospholipid component (MPL) can benefit the GIT through improving barrier function. Our objective was to compare the effects of two digested MFGM ingredients with or without dextran sodium sulfate (DSS)-induced barrier stress on mucin proteins. Co-cultured Caco-2/HT29-MTX intestinal cells were treated with in vitro digests of 2%, 5%, and 10% (*w*/*v*) MFGM or MPL alone for 6 h or followed by challenge with 2.5% DSS (6 h). Transepithelial electrical resistance and fluorescein isothiocyanate (FITC)-dextran (FD4) permeability measurements were used to measure changes in barrier integrity. Mucin characterization was performed using a combination of slot blotting techniques for secreted (MUC5AC, MUC2) and transmembrane (MUC3A, MUC1) mucins, scanning electron microscopy (SEM), and periodic acid Schiff (PAS)/Alcian blue staining. Digested MFGM and MPL prevented a DSS-induced reduction in secreted mucins, which corresponded to the prevention of DSS-induced increases in FD4 permeability. SEM and PAS/Alcian blue staining showed similar visual trends for secreted mucin production. A predictive bioinformatic approach was also used to identify potential KEGG pathways involved in MFGM-mediated mucosal maintenance under colitis conditions. This preliminary in silico evidence, combined with our in vitro findings, suggests the role of MFGM in inducing repair and maintenance of the mucosal barrier.

## 1. Introduction

Mucosal barrier function of the gastrointestinal tract (GIT) is orchestrated by intestinal epithelial cells (IECs), immune cells, and the mucus layer to defend against external stimuli, absorb nutrients, and eliminate waste. The small intestine serves as the main site of the GIT to protect, sense, and respond to external stimuli by means of its physical and molecular components. The physical components of the intestinal barrier are composed of the mucus layer, which acts as the first barrier of defense, and IECs that tightly regulate the paracellular and transcellular passage of luminal contents. The intestinal mucus is made up of high-molecular-weight glycoproteins called mucins, along with lipids, secretory IgG, water, and antimicrobial peptides [1]. Intestinal mucins include both secreted, gel-forming mucins (i.e., MUC2, MUC5AC), which form the protective layer on the epithelial surface, and transmembrane mucins (i.e., MUC3A, MUC1), which are involved in cell signaling [2,3]. Perturbations in the mucosal barrier through various stimuli such as pathogenic infection, dysbiosis of the gut microbiota, various medications, stress, and poor nutrition increase the host’s susceptibility to damage and potentiates the onset of intestinal barrier dysfunction-related diseases, such as inflammatory bowel disease (IBD) and colitis [4]. Furthermore, mucosal barrier dysfunction predisposes individuals to the development of food allergies, celiac disease, and diabetes [5,6]. Hallmarks of gut barrier disruption include increased gut permeability via decreased tight junction protein expression, infiltration of immune cells and their cytokine responses, and decreased mucin through the depletion of goblet cells [6,7]. Once barrier function is compromised, individuals are at heightened risk for insult by further stressors, underscoring the need to find preventative and restorative solutions for adequate barrier function.

Dextran sodium sulfate (DSS) is frequently used as a model for barrier disruption and a colitis-like physiology in both cell culture and in vivo animal models [8,9]. DSS is a water-soluble polysaccharide with a negative charge that is proposed to penetrate IECs, leading to gut leakiness and a subsequent immune response [9]. Compared to other models, DSS is favored for its reproducibility and controllability, serving as a tunable model for severity of inflammation and barrier disruption based on dosage and exposure time [9]. Typical physiological changes that occur after DSS exposure include reduction in tight junction protein expression (i.e., occludins, Zonula occludin (ZO-1), claudins), ulceration, and infiltration of immune cells, mimicking many of the physiological changes that are observed with the pathogenesis of IBD [10,11]. Further characterized by the depletion of goblet cells and the corresponding decrease in mucin production, DSS is associated with a reduction in secreted mucins, including MUC2, which is the predominant secreted mucin in the small intestine and colon. Through various mucin knockout studies and the current evidence on DSS models, the depletion of mucins and changes in their glycosylation patterns are associated with dramatic changes in the gut microbiota and long-term consequences to human health [12,13].

The use of restorative and preventative agents of gut barrier dysfunction, including bioactive nutritional components, have the potential to impact the mucosal barrier through maintenance of its physical and molecular components [14]. Mammalian milk has long been considered a rich consortium of bioactive components, including the milk fat globule membrane (MFGM), which has gained substantial attention in recent years for its copious health benefits. The MFGM consists of a complex tri-layer structure that emulsifies fat globules in milk and is composed of more than 200 different proteins and numerous lipids with unique structural and functional properties [15]. The protein component of the MFGM includes eight major proteins, such as lactadherin (PAS6/7), butyrophilin (BTN), mucin-1 (MUC1), fatty acid binding protein (FABP), mucin-15 (MUC15), xanthine dehydrogenase/oxidase (XDH/XO), adipophilin (ADPH), and hundreds more [15,16]. The predominant lipid components of the MFGM include milk phospholipids (MPL), which consist of phosphatidylcholine (PC; 19–43%), phosphatidylethanolamine (PE; 26–46%), phosphatidylserine (PS; 3–16%), phosphatidylinositol (PI; 1–14%), and sphingomyelin (SM; 18–29%) [17].

Based on in vivo models and clinical trials of infant nutrition, the current evidence supports the role of the MFGM and MPL fraction in gut development [18], brain and cognitive function [19,20], bone growth and remodeling [21,22], cardiometabolic health [23,24,25], and more [26], leading to its incorporation into infant formulas. With respect to its gut-benefiting properties, the MFGM increases the length of the villi of the developing intestine, downregulates pro-inflammatory cytokines, and increases tight junction protein expression [18]. The effects of the MFGM on the gut microbiota are variable. However, there is a consistent finding across studies that it favors increased abundance of bifidobacteria, which are known for their beneficial properties in the gut [26,27]. A potential mechanism of these gut microbiota-related changes upon MFGM supplementation is through regulation of mucin proteins—a key point of interaction for gut microbes. A few studies have previously reported the effects of MFGM on goblet cells and intestinal mucin gene expression [28,29]. Early evidence in short-bowel rat models suggests that the MFGM can increase goblet cell count and the number of MUC1/MUC2-positive cells [28]. Additionally, in an acute colitis prevention mouse model, prophylactic MFGM treatment resulted in an upregulation in *Muc2* and *Muc4* gene expression [29].

As the phospholipid component of the MFGM is often attributed to being the key contributor to MFGM’s health benefits [26], we were interested in first determining whether two MFGM ingredients with varying phospholipid content could influence a variety of mucin proteins (both secreted and transmembrane) using an in vitro digestion approach coupled with intestinal cell co-culture (Caco-2/HT29-MTX). In addition, the current evidence of MFGM on mucin implies the pre-requisite of intestinal stress. Therefore, we also aimed to investigate the preventative role of the MFGM on intestinal mucins with or without challenge using DSS. We used three nutritionally relevant supplementation levels that are translatable to various food formulations (2, 5, 10% (*w*/*v*)) prior to digestion.

## 2. Materials and Methods

### 2.1. In Vitro Digestion of MFGM Ingredients

Two ingredients, a commercial whey-derived MFGM ingredient (~7% phospholipids by weight) and a commercial beta serum powder-derived MPL ingredient (~70% phospholipids by weight), were subjected to simulated digestion following the static INFOGEST 2.0 digestion model and guidelines consisting of oral, gastric, and intestinal phases [30]. MFGM ingredients were resuspended in DI H_2_O at concentrations of 2, 5, and 10% (*w*/*v*) with gentle agitation. For the oral phase, 5 mL of each solution were mixed with 4 mL of simulated salivary fluid, 1.5 mM CaCl_2_, 75 U/mL salivary α-amylase, and DI water up to a final volume of 10 mL. The oral phase was incubated at 37 °C for 2 min with mixing on a rocking platform (VWR, Radnor, PA, USA) at 95 rpm. For the gastric phase, 8 mL of simulated gastric fluid were added to the same tube and the pH was adjusted to 3.0 using 5 M HCl. Then, 2000 U/mL pepsin, 0.15 mM CaCl_2_, and DI water up to a final volume of 20 mL were added and the tubes were incubated at 37 °C for 2 h with mixing. For the intestinal phase, 8 mL of simulated intestinal fluid were added to each tube after the oral and gastric phases and the pH was adjusted to 7.0 using 5 M NaOH. Tubes were incubated with porcine bile salts (10 mM) for 30 min with rocking. Following bile salt incubation, pancreatin (trypsin activity 100 U/mL), 0.6 mM CaCl_2_, and water up to a final volume of 40 mL were added and incubated for 2 h at 37 °C. Preliminary experiments were conducted to determine the appropriate volumes of 5 M HCl and 5 M NaOH for pH adjustments. At the end of the intestinal phase, the resulting digested fluids were immediately centrifuged (15,000× *g*, 4 °C, 45 min) and the micellar fractions, or bioaccessible fractions, were collected from the mid-section of the supernatant with a syringe and filtered through a 0.2 μm nylon filter. Samples were kept frozen at −80 °C until use. All digestion reagents were purchased from Sigma Aldrich (St. Louis, MO, USA).

### 2.2. Caco-2/HT29-MTX Co-Culture

The HT29-MTX-E12 goblet-like cell line and Caco-2 enterocyte cell line were obtained from the European Collection of Authenticated Cell Cultures (Salisbury, UK) and the American Type Culture Collection (Manassas, VA, USA), respectively. Both cell lines were maintained independently in 25 mM high-glucose Dulbecco’s Modified Eagle Medium (DMEM) containing 10% heat-inactivated fetal bovine serum, 1% penicillin–streptomycin (100 units/mL penicillin and 100 units/mL streptomycin), 1% non-essential amino acids (100×), and 1% 200 mM L-glutamine, with media replacement every 48 h. At 80–90% confluency, the two cell lines were passaged and mixed at the physiologically relevant ratio of 90:10 Caco-2 to HT29-MTX cells to mimic the proportions of absorptive and goblet cells found in the small intestine [31]. Co-cultures were plated in 12-well Transwell plates using a seeding density of 4 × 10^5^ cells/insert and incubated at 37 °C in a 5% CO_2_ humidified atmosphere for 21 days post-confluency. The media were replaced with serum-free media one day prior to all experiments. All cell culture reagents were purchased from Thermo Fisher (Gibco, Waltham, MA, USA).

**Figure 1:**
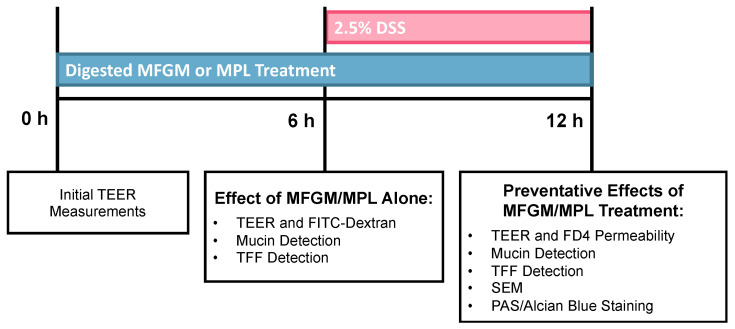
Overview of treatments and measurable outcomes to assess the preventative effects of the milk fat globule membrane (MFGM) and milk phospholipids (MPLs) on dextran sodium sulfate (DSS)-induced mucosal barrier damage using the Caco-2/HT29-MTX (90:10) intestinal cell culture model.

For cell treatment, micellar fractions were diluted to 1:100 in Hank’s Balanced Salt Solution (HBSS; Ca^2+^- and Mg^2+^-free) and then further diluted to 1:10 in serum-free media to maintain the osmotic balance of the cells based on cytotoxicity measurements. Then, 500 μL of the micellar fractions in media were applied to the apical chamber of the cells and fresh serum-free media were applied to the basolateral compartment. After 6 h, cells were either harvested for subsequent assays or further treated with dextran sodium sulfate (DSS) for an additional 6 h to achieve a final concentration of 2.5% DSS in the apical compartment. DSS was applied without removing micellar fraction-containing media to mitigate any effect of washing on the mucosal layer. A graphical representation of cell culture treatments and time points are described in Figure 1.

### 2.3. Transepithelial Electrical Resistance (TEER) and FITC-Dextran Permeability

The transepithelial electrical resistance (TEER) of each well was recorded after washing twice with HBSS (with Ca^2+^ and Mg^2+^) at 0, 6, and 12 h using an EVOM Epithelial Voltohmmeter with an STX2 probe (Sigma). The relative change in TEER value was determined for each well compared to the initial value. Paracellular permeability was determined by measuring the transport of Fluorescein isothiocyanate (FITC)-dextran (FD4; MW = 4 kDa; Sigma) across the cell monolayer in phenol red- and serum-free media. A final concentration of 2 mg/mL was applied to the apical side of the inserts for 2 h at 37 °C in the dark. At the end of incubation, 100 μL aliquots were collected from the basolateral compartment and transferred to black 96-well plates. Fluorescence was measured using a SpectraMaxM2 fluorescent plate reader (490 nm excitation/520 nm emission; Molecular Devices, San Jose, CA, USA) and quantified based on a standard curve of FD4 concentrations ranging from 0 to 2.56 ng/μL.

### 2.4. Slot Blot for Mucin Detection

Cells were washed 2× with PBS and incubated for 30 min on ice with 200 μL RIPA buffer containing complete protease inhibitor (Roche Diagnostics, Indianapolis, IN, USA). Lysates were centrifuged (11,000× *g*, 10 min, 4 °C) and the supernatants were transferred to new 1.5 mL tubes. Protein concentration was determined using the Pierce microBCA kit (Thermo Fisher). For mucin detection, a nitrocellulose membrane (0.45 μm) was pre-wet for 10 min in 1× tris-buffered saline (TBS), and the Bio-Dot SF apparatus (BioRad, Hercules, CA, USA) was assembled according to the manufacturer’s recommendations. After washing each well with 1× TBS buffer, 200 μL of sample (diluted to 17 μg/mL) were loaded per well and pulled onto the membrane via vacuum. The membrane was transferred to a blocking buffer consisting of 3% cold-soluble fish gelatin in TBS buffer for 1 h at 25 °C on a rotating platform (70 rpm). Each membrane was subsequently washed 3× in TBS buffer with 0.05% Tween-20 (TBST) for 5 min. For primary antibody incubation, each antibody was diluted in antibody buffer (AB) consisting of 1% gelatin in TBST and incubated overnight at 4 °C on a rotating platform (70 rpm). The primary antibodies used included: mouse monoclonal MUC5AC (1:5000, Cat. No. WH0004586M7, Sigma Aldrich), mouse monoclonal MUC2 (1:3000, Cat. No. ab11197, Abcam, Cambridge, UK), rabbit polyclonal MUC3A (1:2000, Cat. No. PA5100199, Invitrogen, Waltham, MA, USA), and mouse monoclonal MUC1 (1:3000, Invitrogen, Cat. No. MA1-06503). Following 3× washing with TBST (5 min each), each membrane was treated with 1:3000 Immun-Blot Goat Anti-Mouse IgG-AP Conjugate or Immun-Blot Goat Anti-Rabbit IgG-AP Conjugate (BioRad) in AB and incubated for 2 h at 25 °C with rocking. Membranes were washed again 3× with TBST followed by one wash in TBS, and the AP Color Development kit (BioRad) was used according to the manufacturer’s instructions. Finally, each membrane was washed 2× with DI H_2_O (5 min each) and the membranes were imaged while wet using a BioRad ChemiDoc MP Imaging System.

### 2.5. Scanning Electron Microscopy (SEM)

Cells were washed 2× with PBS and fixed with 3% glutaraldehyde, 2% paraformaldehyde in 0.1 M potassium phosphate buffer (PB; pH 7.2) overnight at 25 °C. Samples were washed with PB for 10 min followed by a single wash with ddH_2_O for 10 min prior to post-fixation with 2% OsO_4_ in ddH_2_O for 30 min. After transferring to a falcon tube, samples were dehydrated using an ethanol series (25% EtOH, 1 × 15 min; 50% EtOH, 1 × 15 min; 70% EtOH, 1 × 15 min; 90% EtOH, 1 × 15 min; 100% EtOH, 3 × 15 min). Finally, after critical point trying, samples were sputter-coated with a 3 nm Pt coating. SEM micrographs were captured using a Hitachi Schottky Field Emission SU5000 Scanning Electron microscope (Hitachi, Tokyo, Japan) at the Molecular and Cellular Imaging Center (MCIC) located at The Ohio State University, Ohio Agricultural Research and Development Center (OARDC) in Wooster, OH, USA. The % coverage was determined using the Fiji software V2.15 [32].

### 2.6. Glycoprotein Staining

Cells were washed 2× with PBS and fixed with 10% neutral buffered formalin. Samples were then sent to the Comparative Pathology & Digital Imaging Shared Resource (CPDISR) facility at The Ohio State University for processing, paraffin embedding, and combined periodic acid Schiff (PAS) and Alcian blue staining. Slides were imaged using an EVOS FL color microscope (Invitrogen) under 40× magnification.

### 2.7. Bioinformatic Analysis

Prediction of MFGM lipid target action was determined by first obtaining the 2D structures of the digestion end products of the three most predominant phospholipid classes from bovine MFGM using PubChem: lysophosphatidylethanolamine 18:1/0:0 (LPE 18:1/0:0); lysophosphatidylcholine 16:0/0:0 (LPC 16:0/0:0); and sphingosine-1-phosphate (S1P) [33,34]. Using these 2D structures, a list of predicted human targets for each PL product was obtained via Swiss Target Prediction v2023 (Supplementary Tables S1–S3). Targets for colitis were obtained from the GeneCards v5.16 and DisGeNet v7.0 databases (Supplementary Table S4) [35,36]. MFGM lipid targets were cross-referenced to colitis targets using a Venn diagram to discern potential targets of MPL for the prevention of DSS-induced colitis (Supplementary Table S5). Kyoto Encyclopedia of Genes and Genomes (KEGG) pathway enrichment analysis of common targets between MFGM lipids and colitis was performed in R using clusterProfiler 4.0 against the *Homo sapiens* genome using *p* < 0.05 as a cutoff criterion (Supplementary Table S6) [37]. The output containing the top 10 pathways ranked by adjusted *p*-value was visualized using a dot plot.

### 2.8. Statistical Analysis

All experiments were conducted at least in triplicate. Data presented are represented as the mean ± SEM where appropriate. Statistical analyses were conducted using one-way analysis of variance (ANOVA) with a post hoc Tukey test for pairwise comparisons or using a Kruskal–Wallis test with a post hoc Dunn’s test where appropriate. Differences of *p* < 0.05 were considered statistically significant. All statistical analysis was performed in GraphPad Prism V10 unless otherwise noted.

## 3. Results

### 3.1. Protective Effects of MFGM and MPL on Caco-2/HT29-MTX Barrier Integrity and Permeability

Caco-2/HT29-MTX (90:10) cells in Transwell plates were treated with digested 0, 2, 5, and 10% MFGM or MPL for 6 h alone (no challenge) or with an additional 6 h 2.5% DSS treatment to induce barrier damage (prevention). The potential of the digested MFGM and MPL ingredients to prevent DSS-induced barrier damage was observed without removing digesta-containing media prior to DSS treatment. The effects of these ingredients on TEER and FD4 permeability were measured under the no-challenge or prevention conditions.

**Figure 2:**
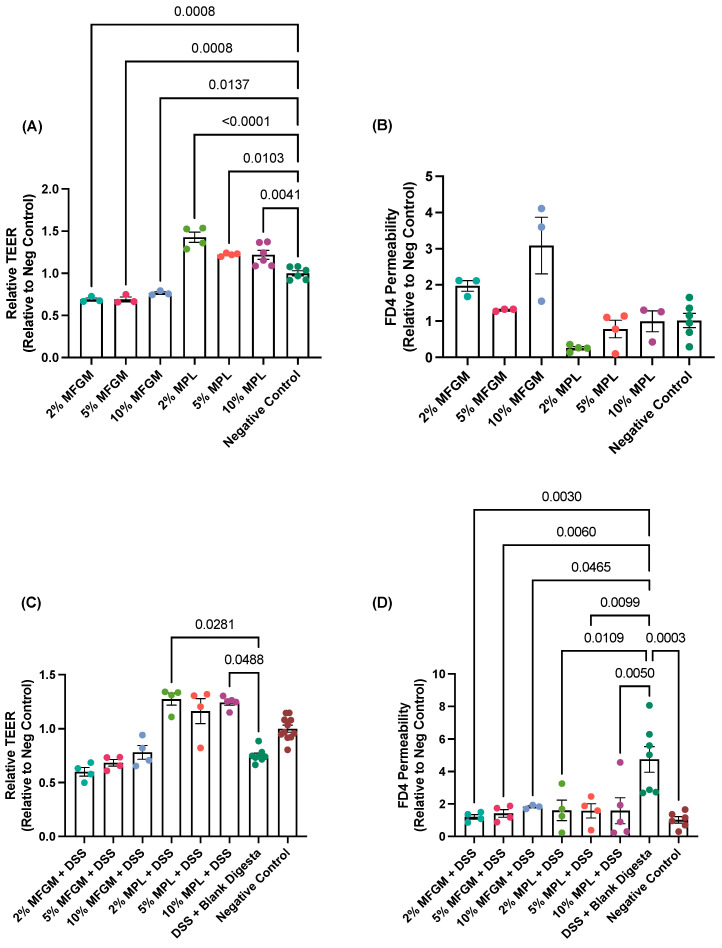
Effects of MFGM and MPL alone (**A**,**B**) or in the context of DSS challenge (**C**,**D**) on transepithelial electrical resistance (TEER) and paracellular permeability of FITC-dextran (FD4). Statistical significance (*p* < 0.05) is denoted for comparisons between negative and/or DSS + blank digesta controls and each treatment using a one-way ANOVA with a post hoc Tukey test (**A**,**D**) and the Kruskal–Wallis test with a post hoc Dunn test (**B**,**C**).

Under no-challenge conditions, the various digested MPL concentrations significantly increased TEER values greater than the negative control, whereas the various MFGM concentrations significantly decreased TEER below the negative control (Figure 2A). Under the FD4 permeability measurement, the MFGM and MPL treatments did not significantly differ from the FD4 permeability of the control (Figure 2B). Changes in TEER and FD4 values are associated with changes in the transport of ions (conductance) and barrier permeability, respectively [38]. Together, our findings indicate the intactness of the cell barrier after digested MFGM or MPL treatments but may suggest possible changes in ion transport through the regulation of tight junction protein expression and/or tight junction pore size, which has been previously reported for MFGM ingredients [39,40].

With the preventative assessment, digested 2 and 10% MPL exhibited significantly higher TEER values compared to the DSS + blank digesta control—a positive control to induce mucosal barrier damage containing both digested water (background digestion elements) and 2.5% DSS (Figure 2C). The same MPL treatments did not significantly differ from the negative control, indicating the prevention of a DSS-induced decrease in TEER by MPL. Despite MFGM showing dose-dependent trends, no significant differences were observed between the MFGM samples and either the DSS + blank digesta or negative controls. For FD4 permeability, all MFGM and MPL concentrations inhibited DSS-induced permeability, suggesting fortification of the mucus layer (Figure 2D).

### 3.2. Effects of MFGM or MPL on DSS-Treated Cells Demonstrated Protective Effects on Mucin Protein Abundance

Given the differences in permeability we observed between digested MFGM/MPL-treated cells with and without DSS challenge, we were interested in observing whether these changes were related to the mucins, as mucin levels are associated with intestinal permeability [41,42]. In general, DSS interferes with standard qPCR reactions [43], so we focused our efforts on characterizing the effects of digested MFGM and MPL on both secreted (MUC5AC, MUC2) and transmembrane (MUC3A, MUC1) mucin protein. MUC5AC and MUC2 were selected, as they represent the predominant secreted mucin for HT29-MTX cells and the predominant human intestinal mucin in vivo, respectively [44,45]. MUC3A and MUC1 were selected, as MUC3A is one of the most well-understood and predominant transmembrane mucin of HT29-MTX cells and MUC1 is known to have aberrant expression in colitis-like phenotypes [44,46].

**Figure 3:**
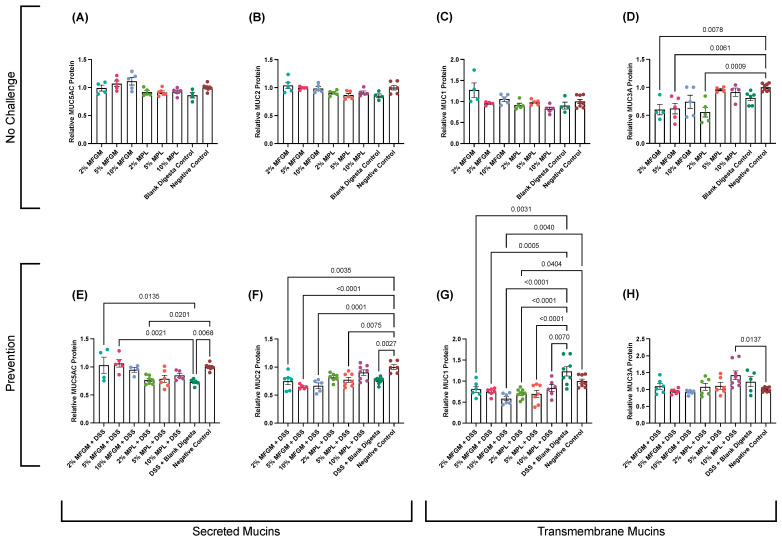
Effects of MFGM and MPL on secreted MUC5AC (**A**) and MUC2 (**B**) and transmembrane MUC1 (**C**) and MUC3A (**D**) in Caco-2/HT29-MTX cells using a slot blot. Prevention of DSS-induced disruption of secreted MUC5AC (**E**) and MUC2 (**F**) and transmembrane MUC1 (**G**) and MUC3A (**H**). Statistical significance (*p* < 0.05) is denoted for comparisons between negative and/or DSS + blank digesta controls and each treatment using a one-way ANOVA with a post hoc Tukey test.

Neither digested MFGM nor MPL treatments had major effects on MUC5AC, MUC2, or MUC1 mucin protein expression without challenge (Figure 3A–C); however, digested 2 and 5% MFGM and 2% MPL led to significant reductions in MUC3A (Figure 3D).

Upon perturbations in the mucosal barrier through DSS challenge, we observed a significant reduction in both secreted mucins, MUC5AC and MUC2 (*p* = 0.0068 and *p* = 0.0027), in response to DSS treatment (Figure 3E,F). The significant reduction in MUC5AC was prevented by all digested MFGM concentrations and digested 5 and 10% MPL (similar to negative control). However, the reduction in MUC2 was partially restored through pre-treatment with 2 and 10% MPL, as these treatments were significantly different with neither the negative control nor the DSS + blank digesta control.

Transmembrane mucin MUC1 is dysregulated in the progression of colitis to colon cancer [47]. In our DSS-induced colitis model, DSS treatment yielded a slight though non-significant increase in transmembrane MUC1 (Figure 3G). However, both MFGM and MPL digesta significantly reduced MUC1 protein levels compared to the DSS + blank digesta control and for some treatments (10% MFGM and 2% MPL) compared to the negative control.

MUC3A was not significantly altered by DSS treatment or the majority of MFGM and MPL digesta concentrations apart from 10% MPL + DSS, which showed an increase in MUC3A (*p* = 0.0137; Figure 3H).

The mucin protein levels under both no-challenge and prevention conditions show similar trends to the data observed in the permeability assessment (Figure 2D) where improvements in DSS-increased permeability are associated with the restoration of secreted MUC5AC levels and, to a lesser degree, MUC2 (Figure 3E,F). Conversely, the absence of weakened permeability in no-challenge conditions (Figure 2B) seems to be associated with no changes in secreted mucins (Figure 3A,B).

### 3.3. DSS-Induced Impaired Brush Border Is Prevented through Digested MFGM and MPL Treatments

**Figure 4:**
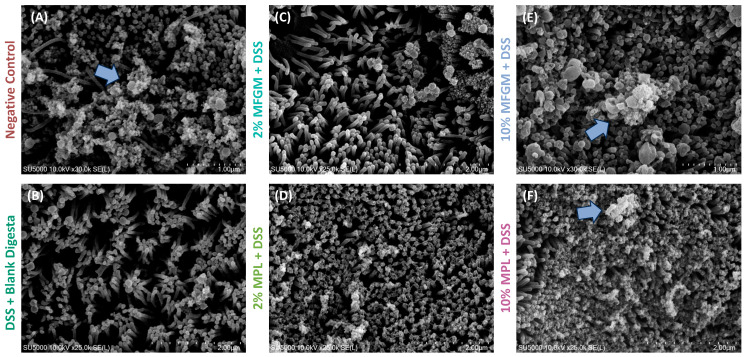
Scanning electron microscopy (SEM) of Caco-2/HT29-MTX cells exposed to digested MFGM or MPL and DSS. (**A**) Negative control with intact brush border and mucosal barrier. (**B**) DSS + blank digesta control with impaired mucosal barrier and brush border. (**C**,**D**) 2% MFGM + DSS and 2% MPL + DSS treatments with partially intact brush border and mucosal barrier after DSS challenge. (**E**,**F**) 10% MFGM + DSS and 10% MPL + DSS treatments with intact mucosal barrier and brush border after DSS challenge. Blue arrow indicates mucin production.

Based on the previous results, we were interested in whether the cell layer structure was different between treatments. To visually observe the effect of MFGM and MPL on cell layer structure, we employed SEM and PAS/AB staining. Cells were once again treated for 6 h with MFGM or MPL and, without removing media, treated with 2.5% DSS for an additional 6 h to induce barrier damage. Only the 2 and 10% concentrations for each ingredient were selected to represent the lowest and highest concentrations, respectively. We observed an intact brush border with mucin production (light blue arrow) for the negative control by SEM imaging (Figure 4A). Treatment with DSS led to impairment of the intestinal barrier through altering surface coverage (Table 1) as well as the absence of mucin-like structures at the cell surface (Figure 4B). Both 2% MFGM and 2% MPL with DSS treatments led to varying degrees of coverage and mucin abundance (Figure 4C,D); however, the 10% concentrations of these ingredients led to nearly complete surface coverage and apparent mucin abundance (Figure 4E,F).

**Figure 5:**
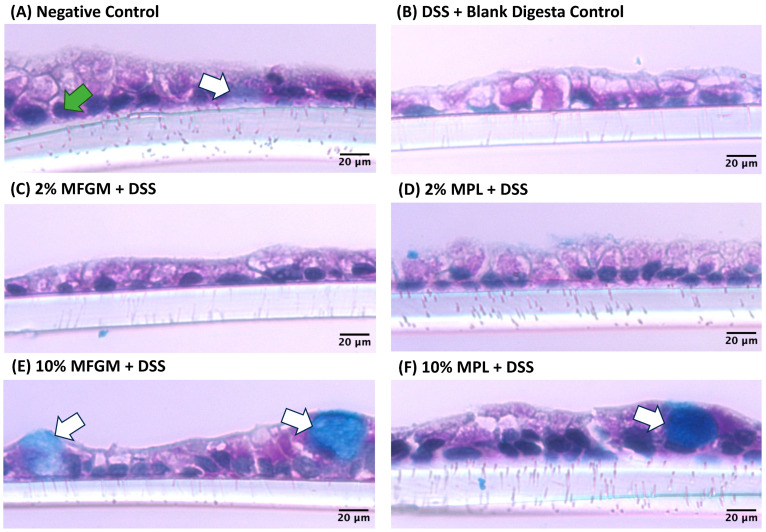
Periodic acid Schiff (PAS) and Alcian blue (AB) staining for neutral and sialylated mucins (pink magenta) and acidic mucins (blue) of Caco-2/HT29-MTX cells exposed to digested MFGM or MPL and DSS. (**A**) Negative control with acidic mucin production barrier. (**B**) DSS + blank digesta control with impaired mucin production. (**C**,**D**) 2% MFGM + DSS and 2% MPL + DSS treatments with partial recovery of mucin. (**E**,**F**) 10% MFGM + DSS and 10% MPL + DSS treatments with acidic mucin production after DSS challenge. The white arrows represent acidic mucin production. The green arrow indicates nuclei.

To further observe whether these effects could be related to changes in mucin production, a cross-section of Caco-2/HT29-MTX cells was stained with PAS and Alcian blue to indicate the presence of neutral mucins and acidic/sialylated mucins, respectively. As shown by the white arrows in Figure 5A, the negative control exhibits both acidic/sialylated and neutral mucins, whereas a depletion of these mucins is observed in the DSS + blank digesta control (Figure 5B). Both digested 10% MFGM and 10% MPL prevent this ablation, as noted by the presence of acidic and/or sialylated mucins (Figure 5E,F) and the 2% concentrations of these ingredients appear to have an intermediary effect (Figure 5C,D).

### 3.4. In Silico Prediction of Pathways Involved in Prevention of DSS-Induced Mucosal Damage by MFGM Lipids

**Figure 6:**
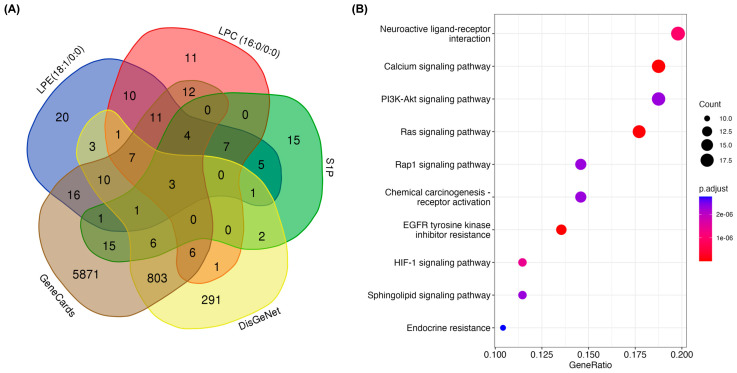
Prediction of pathways involved in prevention of DSS-induced mucosal damage by MFGM lipids. (**A**) Venn diagram of shared and exclusive targets for MFGM lipids (LPE 18:1/0:0, LPC 16:0/0:0, S1P) and colitis gene databases (GeneCards and DisGeNet). (**B**) Dot plot of top 10 enriched KEGG pathways for 100 shared genes between at least one digested MFGM lipid and one colitis gene database list ranked by gene ratio. Gene ratio is the ratio of input of shared genes to the total genes in a given pathway. The dot color indicates adjusted *p*-value.

Regulation of mucin production in intestinal cells is not well understood to date. Therefore, as a beginning insight to explore potential metabolic pathways involved in mucosal barrier function and influenced by MFGM lipids, we implemented a bioinformatic approach. The three predominant digested phospholipid products derived from both MFGM ingredients include LPE (18:1/0:0), LPC (16:0/0:0), and S1P [33,34]. We obtained the potential target genes for these lipids using SwissTargetPrediction and cross-referenced them with colitis-associated genes using GeneCards and DisGeNet databases (Supplementary Tables S1–S4). We observed 100 targets that intersected between at least one colitis target and at least one hydrolyzed phospholipid target (Figure 6A; Supplementary Table S5). Using these targets, a KEGG pathway analysis was performed to determine potential interactions of the MFGM lipids in preventing DSS-induced mucosal damage (Figure 6B). Our in silico findings indicate that the MFGM lipids may be acting through a variety of metabolic pathways involved in cell survival, growth, and responses to changes in the cellular environment, including the PI3K-Akt signaling pathway, Ras signaling pathway, EGFR tyrosine kinase inhibitor resistance pathway, and HIF-1 signaling pathway. Lipid signaling pathways, including sphingolipid signaling and endocrine resistance, appear to also be targets of the MFGM lipids. Notably, the calcium signaling pathway was predicted to be a target of MFGM lipids, which appears to be at the epicenter of most of the predicted target pathways. Intracellular Ca^2+^ is a potent secondary messenger that mediates the crosstalk between signaling pathways [48]. Therefore, investigation into the role of calcium in directing the MFGM lipid response is warranted and may provide further explanation for the role of MFGM lipids in maintaining mucin production, as mucin secretion is Ca^2+^-dependent [49].

## 4. Discussion

Permeability of the mucosal barrier is associated with a reduction in secreted mucins and changes in IECs, among other factors [4,50]. In our study, when comparing permeability and mucin under no challenge conditions, we observed no significant differences between treatments and controls for either FD4 permeability or secreted mucin expression (MUC5AC and MUC2). In contrast, improvements in FD4 permeability were associated with fully or partially restored secreted mucin production in DSS-challenged cells. This relationship suggests the relationship of MFGM or MPL in maintenance of the mucosa under stress conditions and minor effects under non-stressed conditions.

The depletion of goblet cells in DSS-induced colitis and other IBDs is associated with varying changes in mucin proteins. In DSS-induced colitis, MUC1 expression is increased [46] and Muc1−/− mice have lower colitis severity accompanied by an increase in expression of MUC2 and MUC3 in a compensatory manner [51,52]. In our study, we observed an increase in MUC1 protein level in response to the DSS challenge, which was retained to levels of the negative control with MFGM and MPL pre-treatment. Using knockout mouse models with chemically induced colitis, MUC5AC expression has been shown to protect the intestinal barrier through preventing bacterial translocation across the mucosal layer [53]. Similarly, MUC2 knockout mice are more susceptible to the development of colitis [54]. In our study, digested MPL pre-treatment fully prevented DSS-induced reduction in MUC5AC and MUC2 protein levels (Figure 3E,F). We observed a similar trend for digested MFGM, as MUC5AC was maintained after DSS treatment; however, MUC2 protein levels remained at levels similar to the DSS control (Figure 3F). Taken together, digested MPL and MFGM protect against DSS-induced changes in mucin protein in Caco-2/HT29-MTX co-culture. MPL and MFGM have similar, albeit slightly different, effects.

The differences in mucin protein in response to digested MFGM and MPL could be due to the inherent differences in ingredient composition that influence both the relative bioactive lipid purity and digestive processes. The MFGM ingredient contains MFGM proteins, phospholipids (~7 g/100 g powder), whey proteins, caseins, lactose, and more, whereas the MPL ingredient contains a mixture of phospholipids (~70 g/100 g powder), peptides, lactose, and other minor lipid components, and lacks protein entirely. The differences in purity level of phospholipids, and thus the final concentration of phospholipids available to the intestinal cells, could have contributed to the differences observed in mucin protein responses. Despite the differences in phospholipid content between ingredients, we chose to use 2, 5, and 10% (*w*/*v*) solutions at the start of digestion, as these concentrations represent supplementation levels that could be realistically incorporated into foods.

Additionally, the proteins present in the ingredient may have affected the digestive processes and bioaccessibility of MFGM lipids. Glycerophospholipids are hydrolyzed in the sn-2 position by phospholipase A2 (PLA2) in adults and likely by pancreatic lipase-related protein 2 (PLRP2) in infants [55]. It has been suggested that further hydrolysis of lyso-PC and lyso-PE can occur at the mucosal barrier by bile salt-dependent phospholipase B (PLB) in adults [56]. Phospholipid hydrolysis is dependent on dietary components, bile salt concentration, age, and more [56]. For example, protein-rich matrices enhance the bioaccessibility of milk polar lipids and reduce the bioaccessibility of neutral lipids and cholesterol [57]. Additionally, various milk proteins, such as β-lactoglobulin, have been shown to bind milk polar lipids and confer protection from GIT conditions, potentially influencing their fate to mediate cellular metabolic processes [58,59]. These interactions, as well as the action of MFGM-derived peptides, may help explain some of the differences in mucin protein expression between digested MFGM and MPL samples; however, both MFGM and MPL delivered beneficial outcomes on mucin protein.

Previous literature has suggested the role of MFGM and its components in modulation of the mucosal barrier. In an acute colitis mouse model, a prophylactic MFGM treatment followed by DSS increased the gene expression of *Muc2* and *Muc4*, which was associated with the enrichment of nine genera, including *Bifidobacterium* [60]. The MFGM was also found to normalize intestinal development through regulation of villus length and crypt depth, tight junction protein expression, and promotion of goblet cell number using a pup-in-a-cup model [18]. In a similar study investigating the effects of MFGM supplementation in infant formula (IF), MFGM-containing IF restored MUC2 expression to levels of breast milk in a dose-dependent manner in Sprague-Dawley rat pups [61]. Using a piglet model, MFGM supplementation in a basal diet for late gestational pregnant sows resulted in increases in *Muc1*, *Muc2*, *Muc4*, and *Muc13* gene expression in neonatal piglets [62]. Taken together with these studies, our findings support the role of the MFGM and MPL in modification of mucin protein under stress conditions. In general, the modifications of mucin proteins during stress offer a potential explanation for the role of the MFGM in colitis and on the gut microbiota, as changes in mucin protein expression and glycosylation patterns directly influence resident gut microbes [13]. Other critical components of the mucosal barrier, including tight junction proteins and cytokine production, are also positively influenced by MFGM supplementation. The MFGM maintains tight junction protein expression, such as *claudin-4* and *zonula occludin-2*, that aids with the maintenance of barrier integrity in a murine colitis model [39]. The MFGM also reduces the production of several pro-inflammatory cytokines, such as IL-1β and IL-6, to help maintain immune balance [29]. Ultimately, these multiple benefits of the MFGM strengthen its role in the prevention of gut dysfunction-related diseases.

Based on our findings, it remains unclear whether the digested MFGM and MPL components are embedding themselves into the mucosal layer to alter the viscosity of the mucosa and thus inhibit DSS penetration, or whether these components are activating signaling pathways related to mucins. Previous studies have shown the PC can be arranged at the interface between the luminal contents and the mucus layer to confer protection against bacterial translocation [63]. However, the MFGM and its components are vastly complex, which could lead to the activation of a multitude of signaling pathways. We used a predictive approach to identify potential pathway targets for the major digested phospholipid components in the context of colitis and mucin. The top enriched KEGG pathways, such as PI3K-Akt signaling, Ras signaling, HIF-1 signaling, and the EGFR tyrosine kinase inhibitor resistance pathway, are associated with cell survival, proliferation, and differentiation. Jiang and colleagues also identified the PI3K-Akt signaling pathway as a target of the MFGM using immunoblotting techniques [39]. Furthermore, Wu and colleagues used a gene ontology (GO) pathway analysis of differentially expressed genes between DSS control mice and DSS + prophylactic MFGM-treated mice, which also found an upregulation in pathways involved in epithelial cell proliferation [29]. The other enriched pathways in our predictive analysis, including sphingolipid signaling and endocrine resistance, have a direct role in lipid metabolism. At the center of these pathways, Ca^2+^ signaling serves as a potent secondary messenger, coordinating the action of a multitude of pathways [64]. Intracellular Ca^2+^ also regulates mucin secretion. In goblet cells, mucin secretion in response to external and internal stimuli is regulated by intracellular Ca^2+^ levels by which mucin vesicle contents are released from the cell through fusion with the plasma membrane, resulting in up to a 1000-fold volumetric expansion [49]. Based on this preliminary predictive approach and the evidence of the MFGM’s action on mucin protein, this study lays the foundation for future work to investigate the role of Ca^2+^-mediated signaling by the MFGM and MPL to regulate mucin secretion.

## 5. Conclusions

In this study, we examined the effects of two digested MFGM-based ingredients with varying phospholipid content on mucin protein using Caco-2/HT29-MTX cells with or without DSS-induced barrier disruption. Both in vitro digested MFGM and MPL fortified the mucosal barrier to protect against DSS-induced loss in mucin production; however, marginal effects were observed for these ingredients on mucin protein under no challenge conditions. These findings were further corroborated through measurements of FD4 permeability where the MFGM ingredients prevented DSS-induced increases in permeability and by SEM and PAS/Alcian blue staining. Using a predictive bioinformatic approach, these MFGM ingredients appear to influence calcium signaling pathways, which is directly involved in mucin secretion. The findings from this work warrant further exploration of the MFGM to reduce intestinal barrier damage using more complex models such as intestinal organoids that more closely mimic the heterogeneity of the intestinal epithelium and in vivo models, as a major limitation of this study is the lack of interaction with the gut microbiota.

## Figures and Tables

**Table 1:** Surface coverage of intestinal barrier from SEM images after MFGM or MPL treatment and subsequent DSS challenge.

	Negative Control	DSS + Blank Digesta	2% MFGM + DSS	2% MPL + DSS	10% MFGM + DSS	10% MPL + DSS
% Coverage	86.6 ± 2.04	71.2 ± 2.03	78.2 ± 6.74	88.9 ± 0.714	99.3 ± 0.501	99.8 ± 0.087

## Data Availability

The data that support the findings of this study are available from the corresponding author, R.J.-F., upon reasonable request.

## Supplementary Materials

The following supporting information can be downloaded at: https://www.mdpi.com/article/10.3390/nu16070954/s1, Table S1: Swiss target prediction for LPC (16:0/0:0); Table S2: Swiss target prediction for LPE (18:1/0:0); Table S3: Swiss target prediction for sphingosine-1-phosphate; Table S4: Colitis disease targets obtained from the DisGeNet and GeneCards databases; Table S5: Venn diagram categorization of common and exclusive targets; Table S6: KEGG pathway enrichment analysis of common targets shared between MFGM lipids and colitis disease.
